# Large-scale culturing of *Neogloboquadrina pachyderma*, its growth in, and tolerance of, variable environmental conditions

**DOI:** 10.1093/plankt/fbad034

**Published:** 2023-08-09

**Authors:** Adele WestgÅrd, Mohamed M Ezat, Thomas B Chalk, Melissa Chierici, Gavin L Foster, Julie Meilland

**Affiliations:** CAGE—Centre for Arctic Gas Hydrate, Environment and Climate, Department of Geosciences, UiT, The Arctic University of Norway, Dramsveien 201, Tromso 9010, Norway; CAGE—Centre for Arctic Gas Hydrate, Environment and Climate, Department of Geosciences, UiT, The Arctic University of Norway, Dramsveien 201, Tromso 9010, Norway; Department of Geology, Faculty of Science, Beni-Suef University, 24V5+2GF, New Bani Suef City, New Beni Suef City, Beni Suef Governorate 2730401, Egypt; CAGE—Centre for Arctic Gas Hydrate, Environment and Climate, Department of Geosciences, UiT, The Arctic University of Norway, Dramsveien 201, Tromso 9010, Norway; School of Ocean and Earth Science, National Oceanography Centre Southampton, University of Southampton, European Way, Southampton SO14 3ZH, United Kingdom; Aix Marseille Univ, CNRS, IRD, INRAE, CEREGE, Technopole Environnement Arbois-Méditerranée BP 80 13545 Aix-en-Provence, cedex 04 - France; Institute of Marine Research, Oceanography and Climate Research Group, Fram Centre, Hjalmar Johansens gate 14, 9007 Tromsø, Norway; School of Ocean and Earth Science, National Oceanography Centre Southampton, University of Southampton, European Way, Southampton SO14 3ZH, United Kingdom; MARUM—Center for Marine Environmental Sciences, University of Bremen, Leoberner Str. 8, Bremen 28359, Germany

**Keywords:** planktic foraminifera, polar, climate change, marine calcifier, culture experiment

## Abstract

The planktic foraminifera *Neogloboquadrina pachyderma* is a calcifying marine protist and the dominant planktic foraminifera species in the polar oceans, making it a key species in marine polar ecosystems. The calcium carbonate shells of foraminifera are widely used in palaeoclimate studies because their chemical composition reflects the seawater conditions in which they grow. This species provides unique proxy data for past surface ocean hydrography, which can provide valuable insight to future climate scenarios. However, little is known about the response of *N. pachyderma* to variable and changing environmental conditions.

Here, we present observations from large-scale culturing experiments where temperature, salinity and carbonate chemistry were altered independently. We observed overall low mortality, calcification of new chambers and addition of secondary calcite crust in all our treatments. In-culture asexual reproduction events also allowed us to monitor the variable growth of *N. pachyderma*’s offspring. Several specimens had extended periods of dormancy or inactivity after which they recovered. These observations suggest that *N. pachyderma* can tolerate, adapt to and calcify within a wide range of environmental conditions. This has implications for the species-level response to ocean warming and acidification, for future studies aiming to culture *N. pachyderma* and use in palaeoenvironmental reconstruction.

## INTRODUCTION AND BACKGROUND

Planktic foraminifera are single-celled protists that secrete a calcium carbonate shell and are ubiquitous in the upper ocean. *Neogloboquadrina pachyderma* is the dominant species in the polar oceans where it is a key planktic calcifier responsible for up to 25% of the inorganic carbon export above 50° (e.g. Tell *et al.*, 2022). *Neogloboquadrina pachyderma* thrives in a range of environments from upwelling zones to subpolar and polar oceans, including living in waters with temperatures from −2 to +15°C (e.g. Lombard *et al.*, 2009; Zamelczyk *et al.*, 2021) with the Arctic genotype seemingly preferring the lower end of this temperature range (e.g. Darling *et al.*, 2007). It is also able to tolerate a wide range of salinity (30–80) and pH (7.8–8.8) conditions, being found in and near sea ice, brine channels and riverine run-off (e.g. Spindler and Dieckmann, 1986; Manno *et al.*, 2012; Bertlich *et al.*, 2021). The Arctic can be considered a test environment to understand the impact of global warming on marine species due to polar amplification, which means climate change in this region outpaces that experienced by other ocean environments (Miller *et al.*, 2010; Screen and Simmonds, 2010). Ocean acidification (Chierici and Fransson, 2009; Qi *et al.*, 2022), increased sea surface temperature and freshwater dilution from ice melt are impacting the Arctic Ocean rapidly with consequences for the marine ecosystem (Pörtner *et al.*, 2019; Grigoratou *et al.*, 2022). Yet there are large knowledge gaps in understanding polar foraminifera biology, and their response to environmental stressors from changing ocean conditions. For example, asexual reproduction was recently established to be persistent in *N. pachyderma*, having important implications for their survival in the polar oceans by facilitating rapid population growth in spring (c.f., Davis *et al.*, 2020, Meilland *et al.*, 2022, Takagi *et al.*, 2020). Another example of dormancy was reported by Ross and Hallock (2016) in observations of benthic foraminifera, describing its potential link to environmental stressors such as food availability and salinity. In planktic foraminifera, this behaviour has only previously been reported in the Southern Ocean, where *N. pachyderma* hibernates in brine channels in the sea ice near the Antarctic continent (Spindler and Dieckmann, 1986).

Furthermore, understanding *N. pachyderma* life cycle and response to environmental conditions is key to accurately interpret the fossil record and its paleoclimatic implications. *Neogloboquadrina pachyderma* has a long history of use as an environmental indicator species in a fossil assemblage, and its test geochemistry (e.g. isotopic and trace element composition) is widely used for palaeoclimate reconstructions (e.g. Kohfeld *et al.*, 1996; Eynaud, 2011; Ezat *et al.*, 2016; Ezat *et al.*, 2017; Davis *et al.*, 2017a; Schiebel *et al.*, 2018; Livsey *et al.*, 2020). However, a lack of robust calibrations of the relationship between the trace element composition of its test and palaeoenvironmental conditions remains a challenge for polar ocean palaeoceanography. For example, the commonly used Mg/Ca paleotemperature proxy does not have culture-based calibrations for temperatures below 7°C, and trace element proxies for salinity and carbonate chemistry are not well developed in *N. pachyderma* (e.g. Allen *et al.*, 2016; Davis *et al.*, 2017a). This is partially due to difficulties of maintaining planktic foraminifera cultures at low temperature and because of the small size of their tests.

We established a new culture platform at UiT the Arctic University of Norway, Tromsø tailored to culturing polar and subpolar species (for further information, see the ARCLIM website; www.uit.no/project/arclim). In this study, we present the results of a large-scale culturing experiment of *N. pachyderma* (>500 specimens simultaneously in culture) in which variable conditions of low temperature, large salinity gradient and carbonate chemistry (pH, calcite saturation, DIC) could be maintained over weeks. The culturing experiments presented in this article were designed with two objectives in mind: (i) improving our understanding of *N. pachyderma’s* life cycle and tolerance to changing conditions (this study). (ii) To address the lack of proxy calibrations and produce new and improved trace element calibrations specifically optimized for polar conditions (in progress and not presented in this study). These experiments provided us with a unique opportunity to monitor cultured specimens and their biological response to variable and potentially unsuitable water conditions. Culturing experiments, such as this, remain an important tool for improving our understanding of foraminifera, their biology and tolerance to environmental changes.

## METHODOLOGY

### Foraminifera and water sampling

Specimens of *N. pachyderma* (Darling *et al.*, 2006) were sampled from the East Greenland Sea (74.47°N, 01.56°E) using a WP2 plankton net (63 μm mesh, HydroBios), from vertical towing during the CAGE21-2 cruise (Ezat *et al.*, 2021). Small (<100 μm), healthy specimens, identified by their bright orange colour, full cytoplasm, extended and active rhizopodial network, were randomly allocated to 75 mL falcon flasks individually. Specimens were placed into bottles within 30 min after the net recovery to ensure healthy, minimally stressed specimens were picked for the experiments. After 30 min, the net was redeployed to retrieve another batch. Water was collected from the foraminifera collection site in Niskin bottles attached to a CTD (Conductivity Temperature Depth)-rosette and filtered using a 0.2 μm cellulose filter. The water treatments stock solutions were stored in 20 L opaque black jerrycans at < 5°C to limit any biological growth prior to use in the experiments.

### Experimental water manipulation

The water treatments of independently variable conditions (pH, salinity, temperature and barium content) were determined based on field conditions (ambient: 4.5°C, pH 8.1 and salinity 35), realistic past and future Arctic Ocean conditions and a wide range of barium concentrations. These conditions had ranges of temperature 2–7°C, salinity 30–36.7, pH 7.8–8.4 (Table I and Supplementary Table 1) and additional barium in treatments Ba2 and Ba3 with added 1.11 and 1.48 μL BaCl_2_ per 20 L stock solution, respectively.

**Table I:** Water treatment conditions, with treatment labels in parentheses

Water treatment	Low	Med-low	“Ambient”	High
Temperature (°C)	2 (T2)		4.5 (S35)	7 (T7)
Salinity	29.8 (SD29.8)	32.1 (SD32.1) and 33.6 (SI33.6)	35 (S35)	36.7 (SB/SI36.7)
pH (total scale)	7.84 (pH 7.8)		8.10 (pH 8.1)	8.40 (pH 8.4)
Calcite saturation (Ω)	1.7		3.2	5.8
DIC, μmol/kg	2193		2213	2218

Water conditions are manipulated independently with all but one parameter remaining at “ambient” conditions.

The desired salinity was achieved by two different methods for both high and low salinities. First, the two “SI” treatments, SI36.7 and SI33.6, were made by partially freezing filtered seawater at −80°C. The seawater fraction that did not freeze after ~ 10 hr (brine) was used to make the high salinity treatment, and the ice (once melted) was used to make the low salinity treatment to simulate sea ice formation/melt water. Second, the salinity was manipulated by addition of brine produced by evaporation of filtered seawater on a hotplate or distilled water to make the following treatments: SB36.7, SD32.1 and SD29.8. Brine from evaporated seawater (SB36.7) and brine (SI36.7) from frozen seawater, distilled water (SD29.8, SD32.1) and ice meltwater (SI33.6) were added to the ambient (S35) water to create high and low salinities, respectively. Specimens were adjusted to the low salinity of treatment SD29.8 in two steps, initially to SD32.1, then SD29.8 after 24 hr of acclimatization. The two high salinity treatments therefore replicate in terms of salinity, however, the oxygen isotopic and potentially the trace element composition is expected to differ between salinities achieved by freezing and evaporation. Although this is unlikely to impact the life cycle of *N. pachyderma*, the two different approaches are useful for the in-progress geochemical proxy calibrations at UiT.

### Experimental water monitoring and analyses

Water samples from specimen bottles and stock solutions were taken prior to, during and after the experiments to confirm starting water chemistry and monitor any changes during the experiment. Salinity was measured using a WTW Cond 330i conductivity meter, with a precision and accuracy of ±0.05. Samples for determination of carbonate chemistry were performed at the Institute of Marine Research in Tromsø, Norway for total alkalinity (A*_T_*) and dissolved inorganic carbon (DIC) following protocols described in Dickson *et al.* (2007). DIC was determined using a coulometric titration with a Versatile Instrument for the Determination of Titration Alkalinity (VINDTA 3D, Marianda, Germany), and A*_T_* was determined by potentiometric titration in a closed cell on a VINDTA 3S. Routine analyses of Certified Reference Material (SIO, USA) ensured accuracy and precision, both DIC and AT showed a precision of ±2 μmol kg^−1^ (Table I and Supplementary Table 1). From the paired A*_T_* and DIC, salinity and temperature, all other carbonate system parameters (including pH on a total scale and calcite saturation state, Ω) were calculated at *in situ* temperature using CO2SYS software (CO2SYS program, Pierrot and others, 2006). While the analysis was performed at room temperature, calculations were made at experimental temperature using the CO2SYS program to e.g. account for temperature effects on pH (Supplementary Table 1).

The filtered seawater had a pH of ~ 7.9 (total scale). To manipulate its pH, hydrochloric acid (HCl) was added to make a low pH (7.8) treatment, and sodium hydroxide (NaOH) was added to make the high pH (8.4) treatment and the ambient treatments (pH 8.1). Manipulation in this way, using strong acid and base changes pH through changing A*_T_* with little effect on the DIC. Water monitoring during the experiments was done using a Metrohm 914 pH-meter with an Aquatrode Plus with integrated Pt1000 temperature sensor pH-electrode for pH measurements at experimental temperature. The ambient salinity was determined in the field from CTD (Seabird-911) attached to a rosette with 12 Niskin bottles for water sampling of the water column and monitored during the experiments using a portable AdolfR refractometer. Negligent drift (<5%) was observed in the water chemistry (carbonate system or trace element concentrations) of the stock solutions over the course of the experiment.

Water conditions were continuously monitored during the experiments, and depending on water conditions at the previous observation, the water was replaced every 2–6 days to ensure stable conditions. Experiments were ended if conditions became unstable (i.e. pH had drifted >0.05 from the intended value).

To distinguish shipboard and ocean grown foraminiferal test calcite from laboratory grown calcite, all treatment water was labelled (c.f., Bernhard *et al.*, 2004, Evans *et al.*, 2016).

Field light conditions were measured shipboard using a light-probe (Li-cor LI-250 Light Meter) giving values ranging from 30 to 160 photons/sec/cm^2^ over a 24-hour cycle. In the culturing laboratory at UiT, the light conditions in the incubators (Friocell 222 EVO incubators with LED light shelves) were set to replicate the Arctic summer of 24-hour daylight at stable low temperatures, with a lower light intensity during the night, calibrated based on the field measurements. The location of the foraminifera vessels in the incubators was continuously rotated to ensure an equal level of light for each specimen.

### Foraminifera feeding and monitoring

The specimens were fed autoclaved microalgae *Nannochloropsis* (Greco *et al.*, 2020) twice weekly at the start of the experiment and from Day 23 once per week as there was a surplus of food remaining in the culture vessels. A control-batch of specimens was fed freshly hatched *Artemia nauplii*. As there was no apparent difference to foraminifera health between the two food treatments, we opted for feeding with *Nannochloropsis* only for its convenience. The specimens were regularly monitored using inverted microscopes (Zeiss AxioVert.A1, with Filter Set 38, and cameras Axiocam 208 color and 202 mono), noting observations including colour change of cytoplasm, calcification/growth, shell thickening, feeding, rhizopodial activity and gametogenesis (when observed). Size measurements were not always possible, for example due to specimen positioning in the bottles (such as in corners/creases), and the specimen moving during observation causing images to be out of focus and therefore reducing the precision of size measurements.

It was not possible to measure the initial size of the specimens at sea due to time and equipment constraints. Therefore, about 200 specimens of equivalent size to specimens picked for culture (smallest healthy specimens in plankton tow) were picked and preserved in microscope slides on board the R/V Helmer Hanssen for further on shore size analysis. Of these, 40 were measured (umbilical side, longest axis) in the laboratory and these ranged from 51 to 94 μm (std. dev. 11.6 μm), with a mean of 70 μm, and median of 68 μm. We assume the initial size of those foraminifera cultured was therefore ~ 70 μm; however, to ensure we target growth, we consider the in-culture specimen to have grown if their final size is >100 μm. The final size of any given specimen is likely the product of living in conditions allowing for not only survival but also prosperous growth until maturity, as well as the initial health of specimen. This means that specimens in treatments with high mortality, or when treatments ended earlier during the experiment to avoid drift of water conditions, were not allowed to grow to their potential maximum size and are therefore likely smaller and lack crust. Estimated growth per culturing day was therefore calculated to account for the difference in time in culture between the treatments, resulting in a growth estimate normalized for number of days in culture. We also report the growth of 30 asexually produced offspring (proliferation described in Meilland *et al.*, 2022) monitored until 100% mortality, covering a period of ~ 160 days.

Healthy *N. pachyderma* was observed to predominantly attach themselves to the base of the culture-vessels. A specimen was labelled dead when it appeared empty of cytoplasm, with white cytoplasm or a transparent shell, and did not self-attach to the bottle walls. On Day 23, the first experiment was ended (T7) and the remainder by Day 34, except in pH 7.8 where all specimens died in culture, prior to conditions drifting. Some specimens were left to continue to grow under close monitoring if the water conditions were favourable after ending the experiments to see how long they could live in culture.

To measure shell thickness and confirm crust formation in laboratory grown specimens, Scanning Electron Microscopy (SEM) images were taken of a small selection of specimens using a Hitachi TM3030 tabletop Microscope. Images of a total of 10 specimens from all temperature, pH and salinity treatments were made.

### Statistical analyses

Systematic statistical correlation tests were performed on mortality and growth observation data using Excel. A Pearson’s correlation coefficient (R) was calculated to investigate correlation between variability mortality and average final size to different treatments in each category (salinity, pH and Ω, ambient, and temperature) separately. The effects on test size of variable water conditions between treatment categories were compared with test size in ambient water conditions using a two-sample F-test for variances to check the impact the altered water conditions potentially had on growth.

## RESULTS

### Mortality

**Fig. 1:**
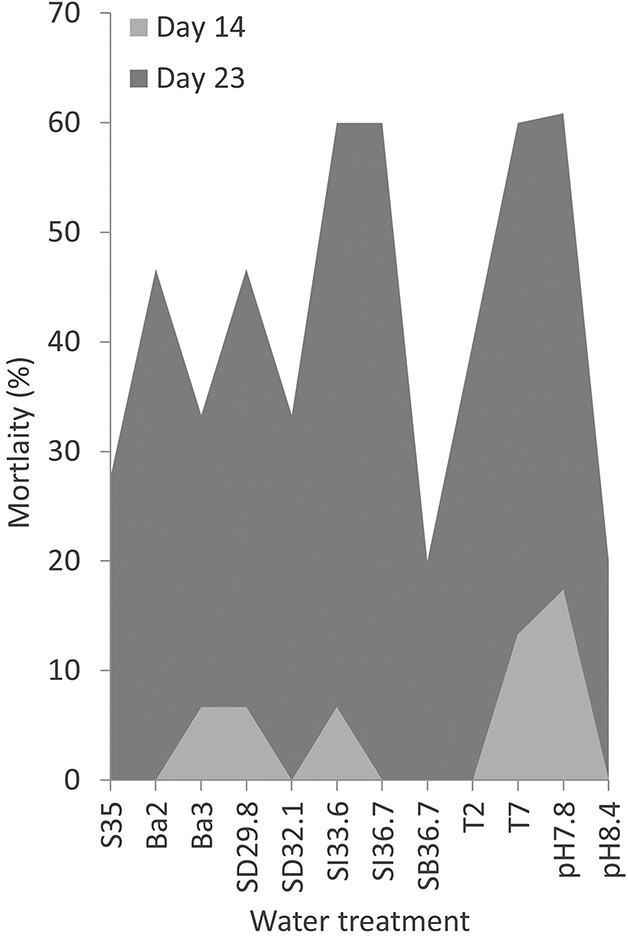
Mortality percentage of *N. pachyderma* per water treatment. Percentage of dead specimen per treatment on Day 14 and prior to ending the first experiment on Day 23. Each treatment initially had 15 specimens, except pH 7.8 with 24 specimens and S35 with 29 specimens.

Mortality remained below 60% across all treatments throughout the experiments (Fig. 1). On Day 7 all specimens were alive, on Day 14 the cumulative mortality was 0–17% with the highest mortalities at low pH (pH 7.8, 17%) and at elevated temperature (T = 7°C, 14%). On Day 23, the lowest cumulative mortality was in the SB36.7 and pH 8.4 treatments (20%) and highest mortality in SI36.7 (60%), under the same salinity conditions as SB36.7. Ba2 had higher mortality (47%) compared with experimental family Ba3 and S35 (ambient) with 27 and 28%, respectively. T2 and T7 displayed similar mortality with 40 and 43%, respectively, and the three low salinity treatments (SD29.8, SD32.1 and SI33.6) had mortalities of 40, 33 and 53%, respectively, on Day 23. The final specimen (for which the experiment was not purposely ended) died on Day 75. Some specimens were left unmonitored after ending the experiments, the last of these specimens (Ba3 n7, SD32.1 n12 and S35 n14) died on Day 77 after collection.

Mortality on Day 23 is not statistically variable between treatments in our ambient or temperature categories (Table II). Perhaps surprisingly, the salinity treatments from the two different approaches of changing salinity resulted in different mortality%. The SI33.6 and SI36.7 treatments have significantly higher mortality% than their (near) equivalents SD29.8, SD32.1 and SB36.7 (Supplementary Fig. 1). While there is no statistically significant correlation to mortality% for the salinity treatments collectively, there is a strong negative correlation (*P* = 0.01) when the SI33.6 and SI36.7 treatments are excluded. The *R*-value for Ω and pH suggests a negative to strong negative correlation, however both with non-significant *P*-values.

**Table II:** Statistical results looking for correlation between % mortality on Day 23 and water treatments

Water treatment	Ambient incl. Ba2 and Ba3	Temperature	Salinity	Salinity excl. ice treatments	Ω calcite	pH
Pearson’s R	−0.04	0.34	−0.02	−0.99*	−0.94	−0.98
p (0.05 conf.)	0.90	0.77	0.97	0.01*	0.22	0.13
*N*	3	3	6	4	3	3

*N* is the number of water treatments compared. *Statistically significant variability. See also Supplementary Fig. 1.

### Inactivity and dormancy

**Fig. 2:**
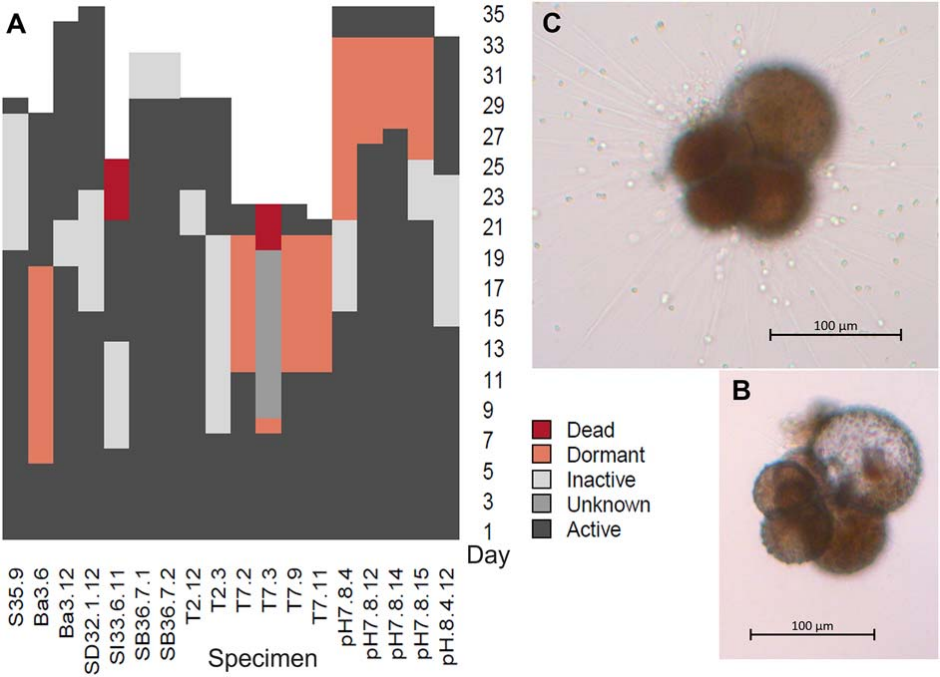
(**A**) Overview of timing of observations of dormancy and activity. White cells mark the end of the experiment, “unknown” indicates the foraminifera had grown but was not observed during the indicated days. (**B**) Specimen pH 7.8 n4 on Day 15 when deemed “inactive” with lumpy pale (white under stereomicroscope) cytoplasm, was not feeding, had low/no rhizopodial activity, and was not sticking to bottle. (**C**) Specimen pH 7.8 n4 on Day 43 when recovered with bright coloured cytoplasm filling the shell, active rhizopodial network, sticking to the bottle sides, and was observed feeding on algae (Nannochloropsis). See also Supplementary Table 2 for dormancy data.

At our sampling location, the healthy/active specimens of *N. pachyderma* were characterized by a bright red/orange cytoplasm, active rhizopodial network and noticeable food intake. In the culture jars, they used the rhizopodial network for feeding, motility and to attach to the walls of the bottles. We define “inactive” specimens as specimens with pale, yellow or light brown coloured cytoplasm, limited/no rhizopodial activity, no feeding observed. “Dormant” specimens are here referred to as appearing with a decaying cytoplasm; apparently empty shell or with grey/white material, with no rhizopodial activity. These specimens also do not stick to the bottle walls (e.g. Fig. 2B vs C). The cytoplasm of dormant specimens is not visible under a stereomicroscope, frequently leading to their labelling as dead. Under the inverted light microscope at higher magnification, the cytoplasm appears blurry or lumpy and pale/brown. Several specimens were therefore initially labelled as dead but later appeared recovered and active again. Inactive and dormant specimen did not add further chambers and were therefore described as not growing.

Several specimens were observed to have periods of inactivity and/or dormancy which lasted from few days to several weeks (Fig. 2). Some of these would go from appearing inactive to dormant then to active again. The “recovered” specimens were healthy and often continued growing. Dormancy could also be inferred when not directly observed, for instance, specimen T7 n3 was observed on Day 8 and described as dormant, presumed dead, and not observed again until Day 20 (dead). Its condition is therefore labelled as “unknown” between these dates, although size measurements indicate growth within this period, so a period of recovery is presumed.

Specimens that were originally labelled dead, which later recovered were often left unmonitored for some days. During this period, other organisms (e.g. diatoms from the genera *Thalassiosira, Pseudo- Nitzschia* and *Chaetoceros*) grew in the bottles without water conditions being strictly regulated, resulting in increased food availability (see section Diet/feeding).

### Shell growth

We observed chamber addition (i.e. growth) in all treatments. Average final test size, as measured by longest axis on the umbilical side, varied widely between treatments (149– 203 μm) with the average smallest specimens found in SI33.6 and largest in Ba3. The average size of specimens from treatments of near-ambient and cold conditions (i.e. S35, Ba2, Ba3 and T2) are largest (~180–200 μm). Treatments with more altered or warmer conditions (low/high pH, Ω, salinity and high temperature) generally had smaller, average size (~150–180 μm), though the *P*-value of this result was not statistically significant using a two-sample F-test (Table III). Chamber addition/growth rate is not linear and varied between individuals. Specimen was observed to go for periods of several days to weeks without adding chambers and/or growing several chambers over a few days. There is no statistically significant variability between final size and treatments in each category (Table III).

**Table III:** Statistical results looking for correlation between final size and growth per culturing days to water treatments

Water treatment		Ambient incl. Ba2 and Ba3	Temperature	Salinity	Salinity excl. ice treatments	Ω calcite	pH
*Final size*	Pearson’s R	0.40	−0.89	0.72	0.50	−0.07	0.08
	p *(0.05 conf.)*	0.74	0.30	0.28	0.31	0.95	0.95
*Growth/culturing days*	Pearson’s R	0.05	−0.28	0.34	0.96*	−0.42	−0.28
	p *(0.05 conf.)*	0.96	0.81	0.51	0.04*	0.72	0.82
	*N*	3	3	6	4	3	3

*N* is the number of water treatments compared. *Statistically significant.

**Fig. 3:**
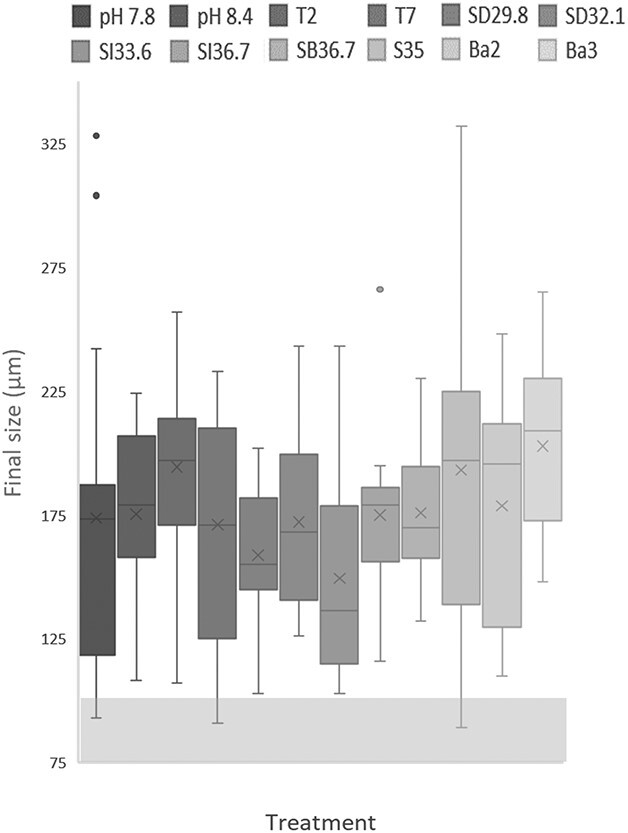
Final shell size range (μm, measured by longest axis) of *N. pachyderma* specimens in each water treatment. The X’s marks the average shell size, and the lines mark the median shell size for each treatment. Separate data points are outlying values. The legend follows the order of the boxes from top left to bottom right. The initial size was 63–100 μm, marked by the striped box at the bottom of the figure.

**Fig. 4:**
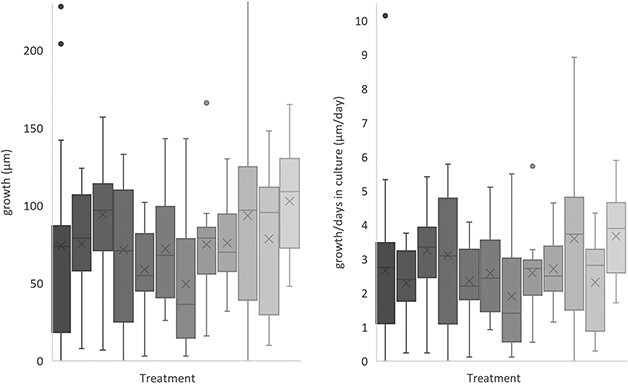
Range of estimated growth (left) and estimated growth per day in culture (right) assuming an initial size of 100 μm for *N. pachyderma* specimens in each water treatment. The X’s marks the average value, and the lines mark the median shell size for each treatment. Separate data points are outlying values. The legend follows the order of the boxes from top left to bottom right. The initial size was 63–100 μm.

Assuming an initial size of 100 μm (see section Foraminifera feeding and monitoring), only three specimens may not have grown (through the addition of new chambers), these were one each in treatments pH 7.8, T7 and S35 and thus this observation is not considered to be related to the culture conditions (Fig. 3). The mean estimated growth is between +49 and +103 μm for all treatments (Fig. 4). The large standard deviations highlight the high intra-treatment variability in size and growth and additional uncertainty from the lack of initial size measurements, particularly for treatments S35 and pH 7.8, which both had more specimens (27 and 24 specimens, respectively) than the other treatments (10–15 specimens each).

For estimated growth per culturing days, the treatments with (near) ambient conditions (except Ba2) have on average more growth per culturing day (3.1–3.7 μm/day) than other treatments (which all grew at < 2.7 μm/day on average). The treatments with least growth per culturing day were SI33.6 and pH 8.4, though we acknowledge this result assumes linear growth with test size. There is a strong correlation between growth per culturing days and salinity (excluding the ice-derived treatments, Table III) with most growth per day in the S35 treatment.

### Chamber formation and specimen fusions

**Fig. 5:**
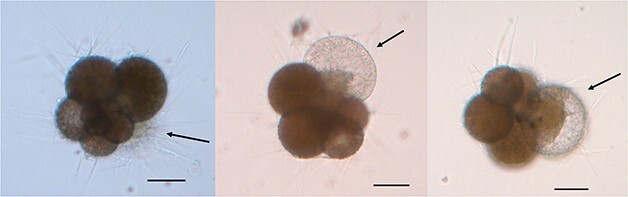
Inverted light microscope image of specimen S35 n4 on Day 20 at 12:20 (left) and at 14:40 (middle) and Day 21 (right). The series show the specimen building a new chamber from the shell filled with bright cytoplasm in 2 hr and 20 min, active rhizopodial network and a budding cyst (left arrow), to the foraminifera building the initial organic lining and crystallizing CaCO_3_ (middle arrow) to cytoplasm spreading into the thickened newly formed chamber (right arrow). The scalebar is 50 μm in all images.

The process of chamber formation from a cyst to initial organic lining and CaCO_3_ crystallizing was observed to last ~ 2 hr in specimen S35 n4 (Fig. 5). Within the 24 hr following the initial calcification, the new chamber was observed to thicken, and cytoplasm extended into the new chamber. Prior to chamber addition, the specimen had extensive rhizopodial networks, brightly coloured cytoplasm filled the shell and a cyst formed at the aperture (as described in Schiebel and Hemleben, 2017). The addition and thickening of a new chamber were observed within 24 hr in several specimens. The final shell thickness measured from SEM images is variable (2–15 μm, including crust) with crust formation observed to occur in specimens from all treatments (Supplementary Fig. 2).

**Fig. 6:**
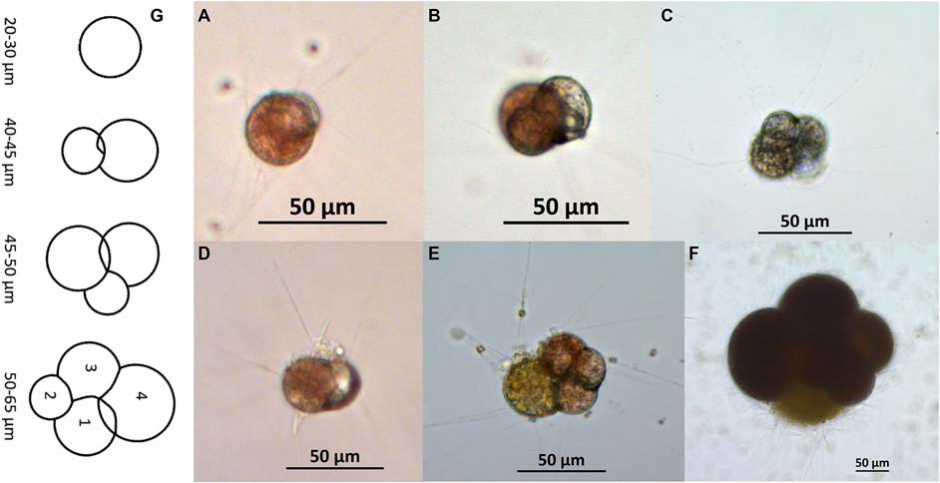
Photos (**A–C**) Offspring 14 on Day 31 with two chambers (A), Day 41 with three chambers (B) and Day 125 with four chambers (C). Photos (**D, E**) Offspring 13 on Day 31 with two chambers (D), Day 41 with four chambers (E), and Day 59, 250 μm measured by longest axis (**F**). Offspring 13 released gametes on Day 78 and measured 330 μm. The scalebars are 50 μm. (**G**) Sketch of chamber addition pattern for chambers one to four (number 1 = proloculus) of the average offspring with the size addition (range, μm) each chamber adds.

Specimens from several treatments were observed to have abnormally shaped chambers and/or growth patterns. For instance, a specimen in treatment S35 added a triangular-shaped chamber (Supplementary Fig. 3). In some large bottles with multiple specimens, we observed specimens attaching to each other, adding additional chambers that “fused” these specimens together creating a “fusion-specimen” (Supplementary Fig. 3). In some bottles where water had not been regularly replaced other photosynthesising organisms such as diatoms from the genera *Chaetoceros*, *Thalassiosira* and *Pseudo-Nitzschia* were observed with associated increases in pH (up to pH = 9) and decrease in DIC caused by biological CO_2_ uptake during photosynthesis. Among three water samples taken prior to pH increasing past 8.5, the average DIC were 2118 μmol/kg (~100 μmol/kg lower than water without diatom growth). The foraminifera specimens in these conditions appeared to thrive (i.e. were feeding, bright cytoplasm, extensive rhizopodia) and grew rapidly, with some not following the classical calcification pattern and rather added chambers in seemingly random locations in sequence resulting in “raspberry-shaped” *N. pachyderma* (Supplementary Fig. 3).

### Growth of the offspring from asexual reproduction of *N. pachyderma*

We observed asexual reproduction from several specimens and were able to observe/monitor growth, mortality and activity of the offspring of specimen M107 (described in Meilland *et al.*, 2022) regularly over time, these observations are described here. Meilland *et al.* (2022) describes the design and purpose of experiments to observe asexual reproduction in *N. pachyderma* and the initial observations of offspring from specimen M107. We also observed gametogenesis in a large portion of the specimen; however we do not have systematic data of gamete release as the specimens were observed 1–2 times per week.

Due to cannibalism (e.g. Meilland *et al.*, 2022), mortality was high until 30 of the 87 offspring were moved to individual 75 mL bottles on Days 22–24, after which mortality dropped. On Day 80, there were still 11 offspring alive, the final specimen died on Day 191.

In total, 20–22 days after they spawned, all offspring had exactly two chambers except for offspring numbers 10 and 29 and offspring 1, 15 and 19, that had one and three chambers, respectively. Four offspring, numbers 6, 20, 21 and 22, comprised two specimens fused together while calcifying (one cell likely having preyed on the other, Supplementary Fig. 3) and had three, two, four and two chambers, respectively.

**Fig. 7:**
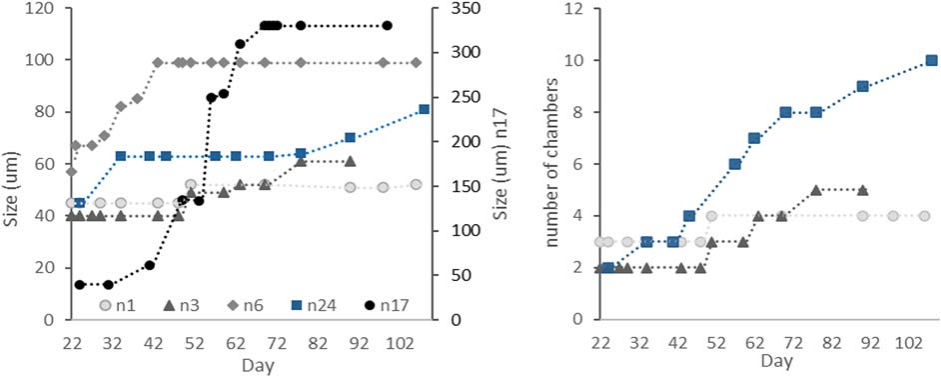
Growth of offspring proliferated from specimen M107 (proliferation described in Meilland *et al.*, 2022). (Left) by size in μm with n17 on the right-hand axis. (Right) by number of chambers. Offspring n1, n3, n6 and n17 follow the classical chamber addition pattern, n24 is a fusion offspring with unusual chamber arrangement hence we record growth in chamber addition, not size. Note that counting number of chambers were not always possible due to the location and orientation of the specimens in the bottles, hence these data are available for a limited number of specimens.

The final offspring sizes were highly variable (Fig. 6), 11 specimens did not grow after being transferred to individual bottles, the smallest specimens were therefore single chambered with a size approximating 30 μm. The largest specimen (number 17, Fig. 7) had a final size of 330 μm. The average final size was 64 μm (4–6 chambers), and 10% of the specimens had a final size >80 μm. Chamber addition was intermittent and highly variable, some specimens added several chambers over 2–3 days and others had longer periods (up to 2 weeks) between chamber additions. Several specimens, still appearing healthy, active, and feeding, did not grow additional chambers during the period of observation (160 days). The longest living specimen (n24, fusion) lived 191 days and had a final size of 81 μm. This specimen added five chambers between Day 34 and 78, however its overall size did not increase due to the unusual chamber arrangement resulting from its fusion (Fig. 7). Offspring 24 did not add chambers after Day 108, although appeared healthy until Day 164 and was found dead on Day 191.

Several specimens grew multiple chambers that were not reflected in the measured size due to chamber arrangement i.e. chambers were added but the measured size did not increase (similar to n24). The plots in Fig. 7 show some specimens that grew by both size and number of chambers. Due to orientation of the foraminifera in the culture bottles, it was often not possible to reliably count the number of chambers, hence we only present this data for three of the offspring.

### Diet/feeding

As described in the Methodology, the specimens for this study were fed with microalgae (*Nannochloropsis,*  Supplementary Fig. 4) alongside a control group being fed with *Artemia* spp. Phytoplankton (e.g. diatoms; *Thalassiosira* spp., *Chaetoceros* spp., *Pseudo-Nitzschcia*) grew in bottles left unregulated and the *N. pachyderma* were observed to feed on these organisms. With sufficient food supply, the specimens were observed to create feeding cysts and/or marine “snowballs” around them (Supplementary Fig. 4) (Fehrenbacher *et al.*, 2018). The offspring from specimen M107 were also observed to feed on each other, noting that this may be a culturing bias resulting from limited space in the well.

## DISCUSSION

### Mortality and growth indicating resilience and ability to adapt

Although we observed large variability in mortality and growth among the treatments and within the treatments, the initial mortality rate was low across all treatments. All specimens were alive after the first week, despite the significant stressors of handling, change of water conditions, moving from the ship to the laboratory and moving into new bottles after returning to the laboratory. After the second week, mortality was still below 20%, showing that *N. pachyderma* is highly resilient to environmental and handling stressors and rapidly changing conditions. Note that all treatments are within realistic past and future living conditions (i.e. were not extreme) for the species *N. pachyderma* in the Arctic Ocean. The high survival rates, despite a rapid change of environment (except SD29.8 where the salinity was decreased in two steps), suggest that *N. pachyderma* does not require a gradual transition to culture treatments when within quasi-natural conditions, as is common procedure in rearing tropical planktic foraminifera (Bijma *et al.*, 1990).

When comparing mortality and final size of the specimen grown in culture, the treatments with the least deviation from the ambient conditions generally had lower mortality and were larger (Figs 1 and 3). This includes treatments S35 (28% mortality on Day 23, mean size: 192.5 μm), Ba3 (that had the largest mean final size, 202.8 μm), T2 and to lesser extent T7 and Ba2 which all had ambient pH and salinity (Table II). This shows that the specimens kept in near-ambient conditions (and with only altered temperature) were minimally impacted by the altered conditions, whereas altering pH, Ω and salinity may have had some negative effects on growth and/or mortality. However, we highlight that there is large individual variability between specimens as is common in biological experiments, and large sample sizes and replicate batches are required to make robust conclusions. Therefore, we here discuss the mortality and growth observations, but refrain from drawing clear-cut conclusions based on our experiments given the relatively small sample sizes involved.

The difference between tolerance to the temperature treatments compared with ambient conditions (4°C) is low (Table II), noting that all temperatures were within known living conditions for *N. pachyderma*. Mortality was marginally higher at 7°C than at 2°C, similarly the estimated growth per culturing day is lower than the ambient for both temperature endmembers although higher than the overall culture average (Figs 3 and 4, Table III) which could be within the noise one could expect from the relatively small number of total specimens.

The low pH and Ω treatment had moderate mortality (43% Day 23) and a relatively small average final size (173 μm vs ambient average of 193 μm). In comparison, the pH 8.4 treatment had lower mortality (20% Day 23) but with a similar average final size (175 μm). The *R*-value (Table II) indicates a strong negative correlation between Ω/pH and mortality; however, the *P*-value is non-significant (*P* = 0.22), potentially due to comparing only three data points (mortality for three treatments on Day 23). Estimated growth per culturing day was similar for the two pH treatments (7.8 and 8.4, Fig. 4). This indicates that the species, although not thriving, is resilient and able to grow and calcify in lower pH conditions and may be relatively resilient to ocean acidification in the Arctic when adequate food is available. However, as the ocean acidifies the difference between the presumed high pH (~9) intracellular calcifying fluid, as seen in other foraminifera species, and the seawater pH and Ω will increase and the foraminifera may require more energy to calcify, resulting in less CaCO_3_ precipitation (De Nooijer *et al.*, 2009). As the results in this study represent growth in size, not amount of calcite precipitated, we are not able to exclude the potential for lower calcite precipitation (e.g. thin and/or low-density shells) at low or high pHs (Ω) in these experiments at this stage.

There is a strong correlation between increasing salinity (excluding ice derived treatments) and decreasing mortality and growth per culturing days, with significantly higher mortality in ice-derived water treatments regardless of water salinity (Supplementary Fig. 1, Tables II and III). The low salinity treatments (SD29.8 and SD32.1) had significantly higher mortality, smaller final size and less growth compared with ambient conditions (S35), with specimens placed in SD32.1 seemingly tolerating the conditions better than in SD29.8. This is in line with findings by Bertlich *et al.* (2021), which indicates that *N. pachyderma*, although it can survive and tolerate low salinity waters, grows less in these conditions. We also note that these two experiments both include additions of distilled water (9–14%), which contains no nutrients, micro-biota or ions and may negatively affect foraminiferal survivability. SB36.7 had the lowest mortality rate in the experiment alongside pH 8.4 (20% Day 23) and moderate mean final size (175 μm). This is largely in line with previous findings (Spindler, 1996; Bertlich *et al.*, 2021) suggesting that *N. pachyderma* is highly tolerant to changes in salinity and can survive in both low and extreme high salinity conditions (such as sea ice brine channels). This also means that they are exposed to a large range of carbonate chemistry with pH ranging from < 8 to > 11 (Fransson *et al.*, 2013). However, in such extreme conditions, the specimens are potentially less active and less likely to add new chambers. The treatments in this study did not reach such extreme conditions as found in brine channels and we still observe chamber addition/growth although to lesser extent and associated with less active specimens than at ambient conditions.

The higher mortality and lower growth in SI33.6 and SI36.7 do not follow the trend of the other salinity treatments (Supplementary Fig. 1). *A priori* we had expected the specimens to do better in the ice-derived water rather than in distilled water, as it is closer to natural seawater in composition. These two treatments were made from the same partially frozen seawater (brine and ice), it is therefore possible that in the process of making this water, it was either contaminated (potential particles in bucket or freezer) or the freezing process changed the water composition to less favourable conditions (e.g. the killing of micro-food sources). However, the proportion of melted ice or brine required to make 20 L of the low/high salinity water treatments is low (10% brine for SI36.5, < 10% ice melt for SI33.5), and therefore it seems unlikely to have had a significant impact on the foraminifera. Given the large variability in mortality and growth within treatments and between replicate treatments as is also observed in tropical species (e.g. Bijma *et al.*, 1990), we suggest future experiments with multiple replicate batches for each experimental water treatment are needed to robustly test the statistical significance of the variance between these two treatments.

As the specimens were observed to feed on a variety of sources (*Nannochloropsis*, *Artemia* spp., cannibalism, detritus, diatoms), it reinforces the suggestion that *N. pachyderma* has an opportunistic feeding behaviour and is omnivorous, feeding on available resources supporting the findings of other studies (e.g. Schiebel *et al.*, 2017; Greco *et al.*, 2019). Specimens growing with a high concentration of other organisms (diatoms of genera *Thalassiosira, Chaetoceros and Pseudo-Nitzschia*) were observed to thrive with active, large rhizopodial networks, colourful cytoplasm and adding chambers rapidly. Additional chambers were often oddly shaped and did not follow the classical pattern in which the specimen(s) initially grew (e.g. “raspberry shaped”). Morphologically abnormal calcification has been linked to stress in some studies (e.g. Caron *et al.*, 1987; Antonarakou *et al.*, 2018; Arenillas *et al.*, 2018), whereas the specimen described in this study was observed to be healthy. As these specimens thrived compared with specimens kept in the pH 8.4 treatment, the increased growth is likely due to the increased food availability and quality (living vs autoclaved organisms), which therefore may have more impact on growth than the water conditions in this culture experiment. Another possibility is that increasing pH via the addition of a strong base is less ideal for calcification than changing the pH via photosynthesis (e.g. the lowering of DIC we record due to the removal of CO_2 (aq)_).

Overall, we argue that *N. pachyderma* is a resilient species, which can adapt to unfavourable and variable conditions although it grows less at lower salinity and pH conditions. In addition, it seems that a large source of fresh food is favourable to their growth. We observed crust formation in all culture treatments, an important observation for understanding its formation and for future geochemical proxy development using laser ablation mass spectrometry, as the species commonly preserves as isolated crust calcite in the fossil record. In addition, the precisely constrained culture conditions mimicking the natural Arctic Ocean, and careful monitoring, allows for accurate culture-based calibration of trace element incorporation to the *N. pachyderma* shell in variable conditions.

### Inactivity and dormancy

Several specimens from multiple treatments and offspring were observed to have periods of inactivity defined as having colourful cytoplasm, no growth, limited/no rhizopodial activity. Others were defined as in a state of dormancy characterized by what appeared to be an empty shell under the stereomicroscope or a shell in which cytoplasm was decaying. The dormant foraminifera were not feeding and exhibited no rhizopodial activity. Foraminifera from both categories could recover to active specimens and continue to grow. With this in mind, we highlight the appearance of dormant specimens under a stereomicroscope (can appear empty/dead) and suggest observing the specimens through an inverted light microscope to confirm the presence of “decaying/pale” cytoplasm not visible under the stereomicroscope or to correctly identify its complete absence (indicating the specimen is dead). Without access to an inverted light microscope (>×5 magnification), we suggest future studies should leave specimens appearing with a pale cytoplasm in its culturing vessel for 7–14 days to allow time to observe potential recovery whilst otherwise maintaining culture conditions. We observed dormancy and recovery in both low pH and high temperature treatments and the ability of specimens to recover supports our assertion that *N. pachyderma* is resilient and able to adapt to changing conditions, confirming observations by Manno *et al.* (2012). This has implications for current and future ocean acidification and warming, with a relatively positive outlook for *N. pachyderma.*

The dormancy and/or inactivity we observe is likely a response to stress where metabolic suppression is used to save energy and survive in unfavourable conditions (Ross and Hallock, 2016; Davis *et al.*, 2017b). *Neogloboquadrina pachyderma* specimens were not observed to feed in this state and will need to recover to an active state at some point to survive and to avoid starvation (c.f., Spindler and Dieckmann, 1986). Natural examples of this state include *N. pachyderma* overwintering in brine channels in Antarctic sea ice (Spindler, 1996). Zamelczyk *et al.* (2021) found overwintering foraminifera under the sea ice, which may have sustained organic matter reservoirs from previous productive season stored in the sea ice. Ross and Hallock (2016) suggest that increased resource availability such as food or light could be potential triggers of recovery in benthic species. In spring, phytoplankton blooms in the Arctic Ocean as the sea ice breaks up, causing increased light availability (e.g. Leu *et al.*, 2015; Trudnowska *et al.*, 2021). *Neogloboquadrina pachyderma* populations also peak in spring (e.g. Reynolds and Thunell, 1986; Jonkers *et al.*, 2010), hypothesized to follow the phytoplankton bloom and increased food availability (c.f., Greco *et al.*, 2019).

Specimens from several treatments (Fig. 2) that endured periods of inactivity and/or dormancy recovered in water with larger concentrations of other organisms i.e. increased food supply with likely increased quality (fresh diatoms). As the light cycle and other conditions remained constant during the experiments, it is likely this increased food availability (and potentially quality with fresh diatoms) that triggered recovery.

The ability of *N. pachyderma* to remain dormant/inactive through the polar winter could allow the species to face short-term unfavourable conditions/disruptions such as freshwater and potentially associated organic matter caused by ice melt and exposure to environmental toxins when the conditions are later favourable. In combination with asexual reproduction (c.f., Meilland *et al.*, 2022), this could facilitate rapid population growth following environmental disruptions. This has implications for ongoing climate change as studies show that earlier sea-ice break-up leads to a mismatch in the timing of phytoplankton and zooplankton blooms (Leu *et al.*, 2015; Ingvaldsen *et al.*, 2021). Studies show a correlation between depth habitat and sea ice proximity, potentially driven by food availability and supported by chlorophyll-α concentrations (Xiao *et al.*, 2014; Rembauville *et al.*, 2016; Greco *et al.*, 2019). If *N. pachyderma* dormancy recovery and population maxima in spring are primarily triggered by primarily food availability, then the species may be able to cope with the de-synchronization of key environmental events. However, if it blooms later than the phytoplankton, an important food source (marine snow, detritus and phytoplankton) will be removed (Carroll and Carroll, 2003; Leu *et al.*, 2015) with consequences for its ability to sustain large populations. To determine the extent, implications and triggers of the dormancy behaviour, further studies about depth habitat and geographic distribution (particular near/in sea ice) and feeding strategies of *N. pachyderma* are necessary.

A consequence of ocean warming is the incursion of subpolar and temperate species into the higher latitudes. This has been observed in the Arctic Ocean where subpolar/Atlantic species become increasingly more common in summer, a phenomenon referred to as Atlantification (e.g. Schiebel *et al.*, 2017; Meilland *et al.*, 2020; Ingvaldsen *et al.*, 2021; Strack *et al.*, 2022). If these species also have the ability to remain dormant in unfavourable conditions, this will allow them to populate the higher latitudes faster as the ocean warms (c.f., Ross and Hallock, 2016). These species could co-inhabit the environment if they all adapt to/thrive in the conditions as intra-species competition does not seem to be an issue among planktic foraminifera (Rillo *et al.*, 2019).

Dormancy as a potential life strategy for *N. pachyderma* also has implications for interpretation of palaeoceanographic records, both with assemblage composition and geochemical based methodologies. As sediment samples often cover hundreds to thousands of years of the geological record, some of the dormancy signal could be minimal compared with the oceanographic variability. However, environmental variability such as orbital changes at high latitudes alter seasonality and could trigger dormancy due to unfavourable conditions more or less frequently during periods of abrupt climate change and such further complicate the recovered climate signal. In addition, during abrupt climate events, the paleo-data signal could be smoothed by the effects of dormancy, and conversely slower climate transitions could be interpreted as abrupt with the appearance of a pseudo-hiatus. However, the part of the *N. pachyderma* population that has a dormant period and then recovers can have grown their initial chambers in a different oceanographic setting to chambers grown after its dormancy (e.g. autumn compared with spring). This would cause substantial differences in the quantitative geochemical signal recovered and should be considered when interpreting palaeoclimate records, both if based on the whole specimen and individual chambers (through chamber amputation or laser-ablation sampling). Analyzing multiple specimens to achieve the population palaeoenvironmental signal, especially in low sedimentation settings that are common across the Arctic basins, may help to minimize this bias.

## CONCLUSIONS

We established a culturing laboratory optimized for polar and sub-polar planktic foraminifera (www.uit.no/project/arclim), with the ability to culture at low temperatures (<1°C) and ≤24-hour light, simulating polar ocean conditions. Here we present methods, observations and results from the first large-scale culturing experiment of *N. pachyderma* where the experiments were designed for geochemical proxy development (the topic of a later study). The specimens were reared over a wide range of conditions: salinity 29.8–36.7, pH 7.8–8.4, Ω: 1.7–5.8, and temperature between 2 and 7°C. Based on our observations of both adult and asexually reproduced specimens, we make the following conclusions:

- Growth (chamber addition and size increase) rate and patterns are highly variable between individuals (adults and offspring) even in identical/equivalent conditions for the species in line with findings for tropical foraminiferal species. Future studies should consider having multiple replicate batches, each comprising multiple specimens, to draw robust conclusions about mortality and growth in relation to environmental conditions.
- *Neogloboquadrina pachyderma* is resilient, tolerate variable environmental conditions and can recover from unfavourable conditions. The species can survive in a large range of environmental conditions, including sudden changes to its environment, and uses dormancy as a way to cope with short-term exposure to unfavourable conditions. However, the specimens, on average, had shorter lifespans and grew less in conditions (pH/Ω, temperature and salinity) deviating substantially from the field collection site in the Greenland Sea, particularly low pH and Ω and salinity conditions.
- We observed multiple specimens entering periods of dormancy and/or inactivity and later recovering and some continuing growing. Dormancy in *N. pachyderma* could have implications for population recovery after environmental disruptions such as a sudden meltwater pulse and seasonal (winter) conditions. Food (fresh and alive) could be a possible trigger for recovery, correlating to such events as the spring bloom of phytoplankton. The presence of dormancy in the fossil record may complicate interpretation of the paleoclimate record as it could both smoothen and amplify a given climate signal.

## Supplementary Material

Supplementary_Table_1_fbad034

Supplementary_Table_2_fbad034

Sup_Fig_1_fbad034

Sup_Fig_2_fbad034

Sup_Fig_3_fbad034

Sup_Fig_4_fbad034

## Data Availability

Data are available on request to the main author until publication on the Cristin database (https://www.cristin.no/).
