# Supplementary material for: Large-scale culturing of Neogloboquadrina pachyderma, its growth in, and tolerance of, variable environmental conditions
Source: J Plankton Res. 2023 Aug 9;45(5):732–45. doi: 10.1093/plankt/fbad034 (PMC10539212; doi:10.1093/plankt/fbad034)
Supplement: Supplementary_Table_1_fbad034 [file supplementary_table_1_fbad034.docx]

**Supplementary Table 1.** Detailed overview of culture water treatments, their respective conditions, and the number of specimens in each treatment.

| **Water treatment** | **T2** | **T7** | **S35** | **SD29.8** | **SD32.1** | **SI33.6** | **SI/**  **SB36.7** | **pH7.8** | **pH8.4** | **Ba2** | **Ba3** |
| --- | --- | --- | --- | --- | --- | --- | --- | --- | --- | --- | --- |
| Temperature (°C) | 2 | 7 | 4.5 | 4.5 | 4.5 | 4.5 | 4.5 | 4.5 | 4.5 | 4.5 | 4.5 |
| Salinity | 35 | 35 | 35 | 29.8 | 32.1 | 33.6 | 36.7 | 35 | 35 | 35 | 35 |
| pH  (Total scale) | 8.10 | 8.12 | 8.10 | 8.12 | 8.12 | 8.14 | 8.05/8.07 | 7.8 | 8.4 | 8.1 | 8.1 |
| Calcite Saturation (Ω) | 3.2 | 3.2 | 3.2 | 2.5 | 2.8 | 3.14 | 2.9/3.0 | 1.7 | 5.8 | 3.2 | 3.2 |
| Dissolved Inorganic Carbon (DIC, µmol/kg) | 2213 | 2228 | 2213 | 1903 | 2039 | 2156 | 2330/2267 | 2193 | 2218 | 2213 | 2213 |
| Barium concentration (µmol/kg) | 2x *in situ [Ba]* | 2x *in situ [Ba]* | 2x *in situ [Ba]* | 2x *in situ [Ba]* | 2x *in situ [Ba]* | 2x *in situ [Ba]* | 2x *in situ [Ba]* | 2x *in situ [Ba]* | 2x *in situ [Ba]* | *3x in situ [Ba]* | 4x *in situ [Ba]* |
| Number of specimens in culture | 29 | 15 | 15 | 15 | 15 | 15 | 15 | 23 | 15 | 15 | 15 |
