# Supplementary material for: Large-scale culturing of Neogloboquadrina pachyderma, its growth in, and tolerance of, variable environmental conditions
Source: J Plankton Res. 2023 Aug 9;45(5):732–45. doi: 10.1093/plankt/fbad034 (PMC10539212; doi:10.1093/plankt/fbad034)
Supplement: Supplementary_Table_2_fbad034 [file supplementary_table_2_fbad034.docx]

**Supplementary Table 2.** Overview of specimens that went through stages of inactivity and/or dormancy and day of recovery. The data shows specimens entering inactivity or dormancy at varying life stages and a variable number of days spent in the dormant/inactive stage.

| **Water treatment** | **Specimen #** | **Day inactive** | **Day dormant** | **Day active** | **# of days to recover** |
| --- | --- | --- | --- | --- | --- |
| **S35** | 9 | 20 |  | 29 | 9 |
| **Ba3** | 6 |  | 7 | 19 | 12 |
|  | 12 | 19 |  | 22 | 3 |
| **SD32.1** | 12 | 16 |  | 24 | 8 |
| **SI33.6** | 11 | 7 |  | 13 | 6 |
| **SB36.7** | 1 | 30 |  | 33 | 3 |
|  | 2 | 30 |  | 33 | 3 |
| **T2** | 12 | 21 |  | 24 | 3 |
|  | 1 | 8 |  | 21 | 13 |
| **T7** | 2 |  | 12 | 21 | 9 |
|  | 3 |  | 8 | Grew between day 8 and 20 | N/A |
|  | 9 |  | 12 | 21 | 9 |
|  | 11 |  | 12 | 21 | 9 |
| **pH7.8** | 4 | 15 | 22 | 34 | 12 |
|  | 12 |  | 27 | 34 | 7 |
|  | 14 |  | 25 | 34 | 9 |
|  | 15 | 22 | 25 | 34 | 9 |
| **pH8.4** | 12 | 15 |  | 25 | 10 |
