# Supplementary figures and images for: Large-scale culturing of Neogloboquadrina pachyderma, its growth in, and tolerance of, variable environmental conditions
Source: J Plankton Res. 2023 Aug 9;45(5):732–45. doi: 10.1093/plankt/fbad034 (PMC10539212; doi:10.1093/plankt/fbad034)

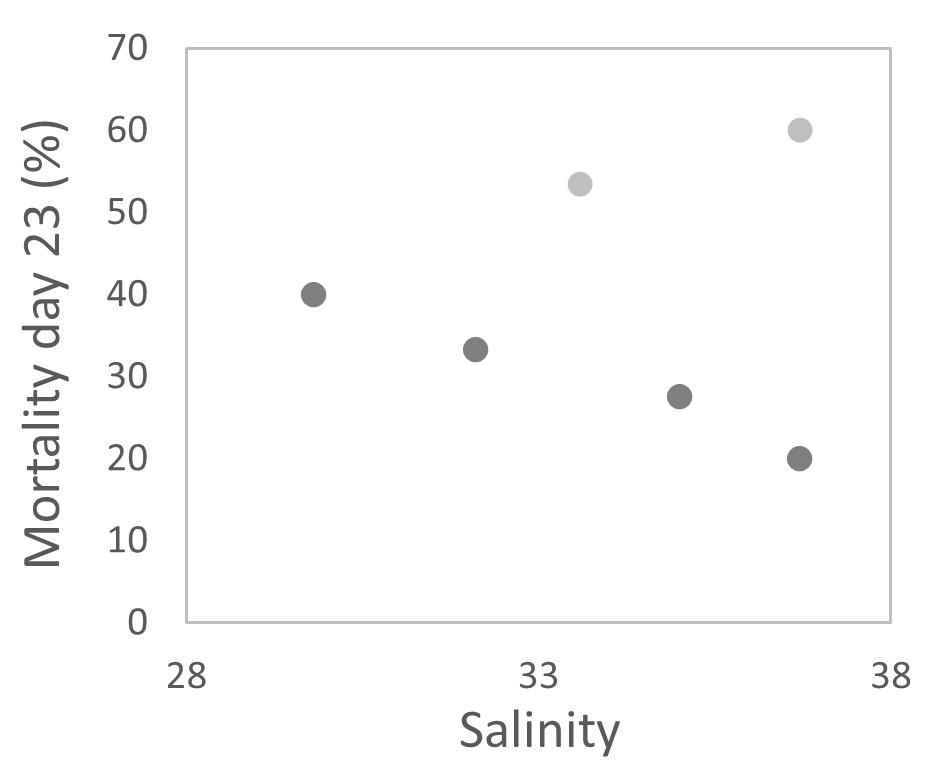

Supplement: Sup_Fig_1_fbad034 [file sup_fig_1_fbad034.jpeg]

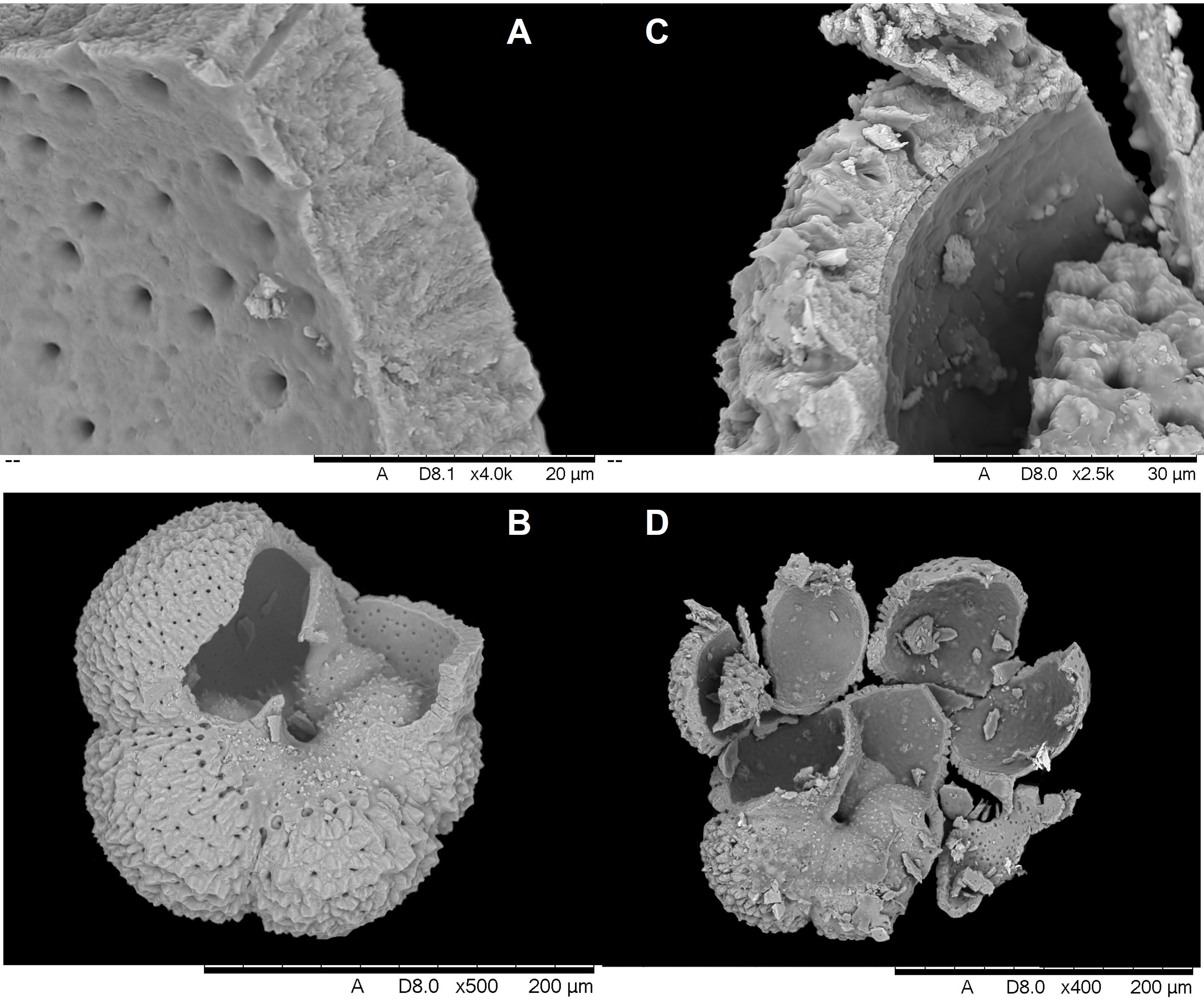

Supplement: Sup_Fig_2_fbad034 [file sup_fig_2_fbad034.jpeg]

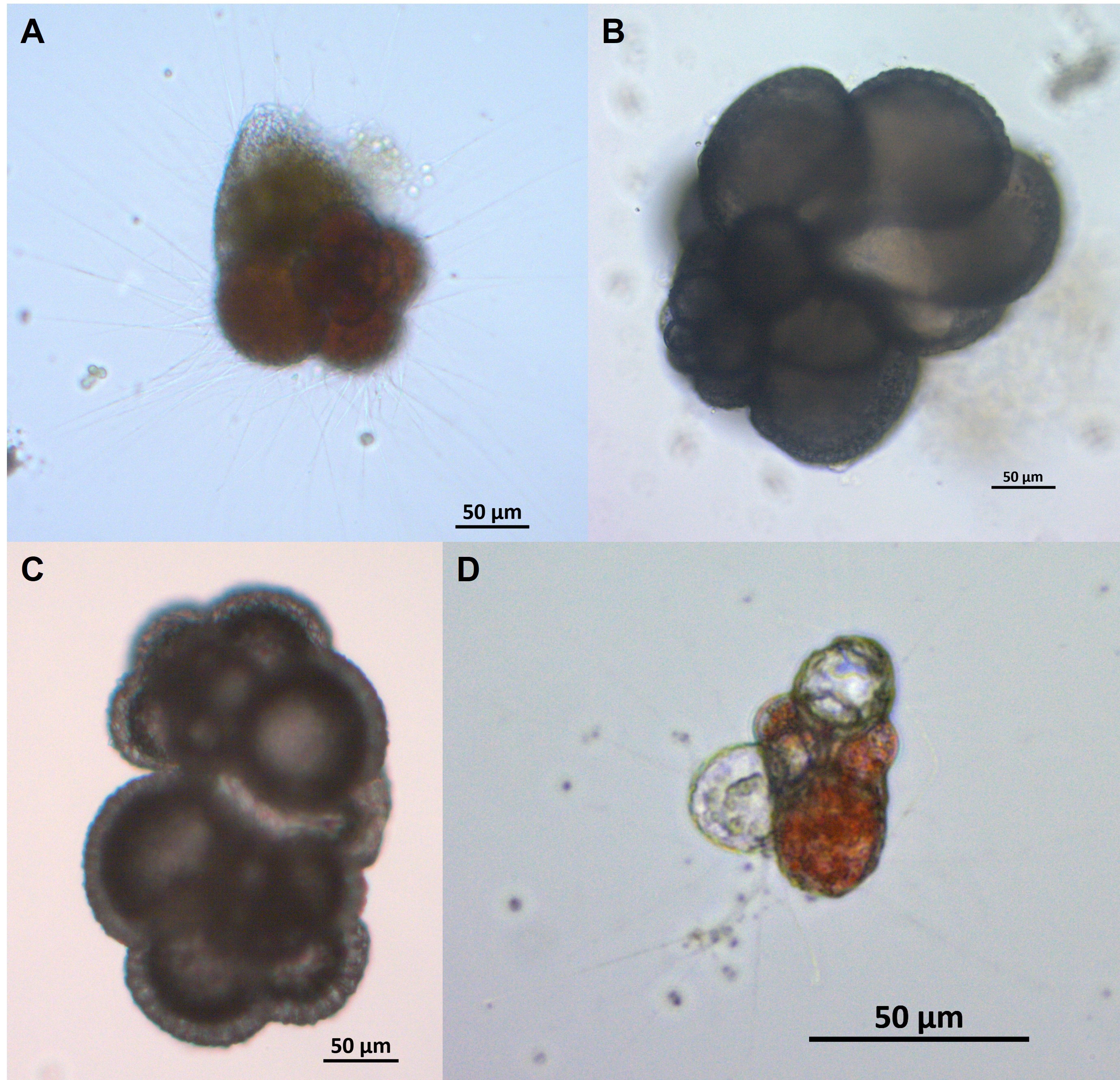

Supplement: Sup_Fig_3_fbad034 [file sup_fig_3_fbad034.jpeg]

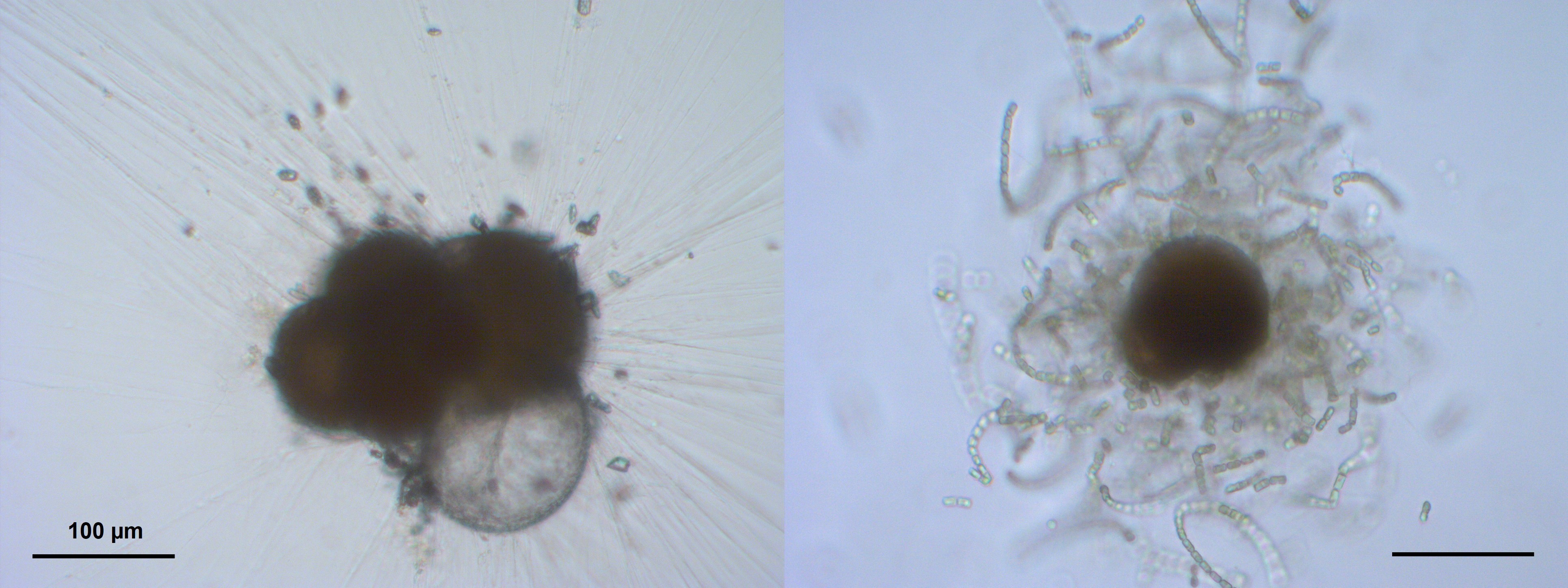

Supplement: Sup_Fig_4_fbad034 [file sup_fig_4_fbad034.jpeg]
